# Which drinkers have changed their alcohol consumption due to energy content concerns? An Australian survey

**DOI:** 10.1186/s12889-022-14159-9

**Published:** 2022-09-19

**Authors:** Jacqueline Bowden, Nathan J. Harrison, Joanna Caruso, Robin Room, Simone Pettigrew, Ian Olver, Caroline Miller

**Affiliations:** 1 National Centre for Education and Training on Addiction (NCETA), Flinders Health and Medical Research Institute, Flinders University, GPO Box 2100, Adelaide, SA 5001 Australia; 2 Health Policy Centre, South Australian Health and Medical Research Institute, Adelaide, SA Australia; 3 Centre for Alcohol Policy Research, School of Psychology & Public Health, La Trobe University, Bundoora, VIC Australia; 4 Department of Public Health Sciences, Centre for Social Research On Alcohol and Drugs, Stockholm University, Stockholm, Sweden; 5 The George Institute for Global Health, University of New South Wales, Sydney, NSW Australia; 6 The National Drug Research Institute, Curtin University, Bentley, WA Australia; 7 School of Psychology, The University of Adelaide, Adelaide, SA Australia; 8 School of Public Health, The University of Adelaide, Adelaide, SA Australia

**Keywords:** Alcohol drinking, Energy intake, Weight gain, Obesity, Survey studies

## Abstract

**Background:**

Alcohol is a discretionary, energy dense, dietary component. Compared to non-drinkers, people who consume alcohol report higher total energy intake and may be at increased risk of weight gain, overweight, and obesity, which are key preventable risk factors for illness. However, accurate consumer knowledge of the energy content in alcohol is low. To inform future behaviour change interventions among drinkers, this study investigated individual characteristics associated with changing alcohol consumption due to energy-related concerns.

**Methods:**

An online survey was undertaken with 801 Australian adult drinkers (18–59 years, 50.2% female), i.e. who consumed alcohol at least monthly. In addition to demographic and health-related characteristics, participants reported past-year alcohol consumption, past-year reductions in alcohol consumption, frequency of harm minimisation strategy use (when consuming alcohol), and frequency of changing alcohol consumption behaviours because of energy-related concerns.

**Results:**

When prompted, 62.5% of participants reported changing alcohol consumption for energy-related reasons at least ‘sometimes’. Women, those aged 30–44 years, metropolitan residents, those with household income $80,001–120,000, and risky/more frequent drinkers had increased odds of changing consumption because of energy-related concerns, and unemployed respondents had reduced odds.

**Conclusions:**

Results indicate that some sociodemographic groups are changing alcohol consumption for energy-related reasons, but others are not, representing an underutilised opportunity for health promotion communication. Further research should investigate whether messaging to increase awareness of alcohol energy content, including through systems-based policy actions such as nutritional/energy product labelling, would motivate reduced consumption across a broader range of drinkers.

## Background

The substantial consequences associated with alcohol use are well-recognised, and are particularly pertinent in countries with high per capita consumption, including Australia [1]. Worldwide in 2016, an estimated 3 million deaths (5.3% of all deaths) and 5.1% of all disability-adjusted life years lost (132.6 million) were from alcohol use [2]. Current evidence supports causal relationships between alcohol consumption and a range of health conditions, including at least seven types of cancer [3]. Importantly, alcohol is also implicated in weight gain, which may interact with alcohol use itself to exacerbate risk of adverse health outcomes including some cancers. Australian overweight and obesity rates have steadily increased from 57% in 1995 to 67% in 2017–18 [4, 5] and now account for 8.4% of total disease burden; of all modifiable risk factors, only tobacco use makes a greater contribution to this burden [6].

### Alcohol consumption and associations with energy intake

In cross-sectional studies, higher alcohol consumption is associated with higher body mass index (BMI) [7], overweight and obesity [8], and increased risk of weight gain [9]. For those above normal weight range, odds of lifetime alcohol use disorders were increased in United States national data [10]. Increased alcohol consumption was also associated with weight gain during COVID-19 restrictions [11]. It should be noted however that due to increases in lipid oxidation and energy expenditure, chronic alcohol use disorder has been associated with lower body weight [12]. Taken together, these findings highlight weight gain risk for the population majority consuming moderate alcohol amounts, rather than only at an extreme of consumption.

Alcoholic drinks are the main contributor to discretionary energy intake for Australian adults aged over 19 years, with this proportion increasing for some age groups (e.g., those 51 years and over) in recent decades [13]. Alcohol is also a major contributor to total energy intake. For those reporting recent alcohol consumption in Australian population survey data, alcoholic beverages contributed 16% of total daily energy intake [14], far exceeding Australian advice that alcohol should contribute no more than five percent of total intake [15].

Whilst pure alcohol is energy-dense, the carbohydrate content of alcoholic beverages also contributes to energy intake. In particular, wine can contribute considerably to dietary free sugar, although there is wide variation in carbohydrate and energy content between specific products [16]. Further, energy consumed from food is increased after the consumption of alcoholic compared to non-alcoholic beverages [17]. Thus, population reductions of alcohol consumption may also reduce rates of overweight and obesity.

### Motivations to reduce alcohol consumption, and awareness of energy content

‘Health reasons’ including weight often motivate both reduced alcohol intake [18] and non-drinking [19]. Risky drinkers in English population data most frequently reported improved fitness, weight loss, and future health problems as reasons for reduced consumption [20]. While this suggests that general health concerns may result in reduced alcohol consumption, specific motivations to avoid energy intake via reductions in alcohol use are not well understood. Methods of avoiding energy intake could be either minimising alcohol intake when drinking (i.e., by engaging with harm-reduction strategies) or reducing overall alcohol intake.

Current evidence indicates low community awareness of the energy content of alcohol across Europe and the United States with a recent meta-analysis finding only 26% of participants (95% confidence interval [CI] = 18–36%) accurately estimated energy content of alcoholic beverages [21]. In New Zealand-based research, 65% of participants underestimated the energy content of alcoholic beverages in the absence of specific energy labelling [22]. Energy content has also been noted as ‘surprising’ by focus group participants [23].

### Aims of the present study

There is currently limited evidence on energy-related concern and its association with changed drinking behaviour. Therefore, it is important to investigate characteristics of drinkers who may already be changing behaviours because of energy-related concerns, or may not be and require increased focus, to determine priority target groups for future interventions.

This survey study aimed to investigate changing alcohol consumption behaviours resulting from energy-related concerns. Further, it aimed to study the demographic and alcohol consumption-related characteristics associated with changed alcohol intake due to energy-related concerns.

## Methods

### Design

Online survey data were collected in Australia during October 2017, as part of a broader study of attitudes towards alcohol consumption in the presence of children [24]. Eligible participants were adults aged 18–59; across Australia, the legal age for alcohol purchase or consumption on licensed premises is 18 years. Potential participants across each Australian state and territory who were already registered with a survey provider (i.e., who had previously been recruited for survey research, and had provided basic demographic details to facilitate invitation to other research for which they may be eligible) were recruited via an email invitation. Initial screening items were used to confirm participant eligibility and representation by key demographic quotas (i.e., approximately equal male–female ratio, ≥ 50% parents) within a total pre-specified sample of *N* = 1,000. Once a quota had sufficient representation, additional participants within the quota could not proceed with completing the full survey.

Only data from those who regularly consumed alcohol were retained for the present analyses; of the 1,000 initial respondents, 197 (19.7%) who did not report past-year alcohol consumption at least monthly were excluded. Open-text comments from a further two (0.2%) suggested non-genuine responses, so all data from these respondents were also excluded. Therefore, the final sample comprised 801 participants.

### Procedure

Participants were offered redeemable points from the survey provider (e.g., for gift cards or charitable donations) equivalent to < AUD$5. The provider satisfied International Organization for Standardization standards (AS ISO 26362).

### Measures

#### Demographic and health-related characteristics

Participants reported gender, age, marital status, completed education, current paid employment status, annual household income (AUD$, pre-tax), and parental status (living with child/children under 18 years). On the basis of respondent postcode, remoteness (major cities vs regional/remote areas, according to Australian Statistical Geography Standards [25]) and general relative socioeconomic disadvantage (collapsed to most disadvantaged [quintiles 1–2] vs least disadvantaged [quintiles 3–5] using the Index of Relative Socioeconomic Disadvantage 2016, a composite area-level measure based on 16 individual- and household-level variables in population data, e.g., household internet connectivity and occupation classifications [26]) were also derived.

BMI (kg/m^2^) was calculated from height and weight, reported in participants’ preferred (metric/imperial) units; 153 (19.1%) reported they ‘did not know’ or ‘could not say’ their height (*n* = 119, 14.9%) and/or weight (*n* = 125, 15.6%). Improbable low outlier BMI values more than three times below interquartile range (≤ 11.1, *n* = 6) were excluded. Participants also reported perceived weight on a single item (“Do you consider yourself to be…?”) with response options ‘Underweight’, ‘An acceptable weight’, or ‘Overweight’, plus ‘Don’t know/Can’t say’.

#### Alcohol consumption characteristics

Per population surveys [18], standard graduated frequency items accompanied by an image of the number of standard drinks in typical servings of various beer, wine, and spirit beverages were used to assess alcohol use [27]. Past-year average daily consumption of > 2 standard drinks (each containing 10 g/12.7 mL pure alcohol) indicated long-term high-risk consumption above the threshold in national (NHMRC) guidelines [27]. Revised NHMRC guidelines were released in December 2020. However, the 2009 long-term guideline (“For healthy men and women, drinking no more than two standard drinks on any day reduces the lifetime risk of harm…”) was current during data collection, and therefore used to assess patterns of drinking known to put health at risk at this time ([27] p. 3). As opposed to the 2009 single occasion/short-term risk guideline, the long-term guideline acknowledged cumulative longer-term effects of alcohol consumption, including a potential association with overweight and obesity.

Past-year frequency of alcohol consumption was based on single item “In the last 12 months, how often did you have an alcoholic drink of any kind?”, with six frequency levels (e.g., Every day, 5 to 6 days a week) collapsed to ‘Daily or weekly’ vs ‘Monthly’.

To measure current alcohol-related risk perceptions, participants estimated low-risk levels using a single item for each of adult males and females (i.e., How many “standard drinks” do you believe an adult male/female could drink every day for many years without adversely affecting his/her health?) [18], with a ‘Don’t know’ option (47.1% and 53.2% for males and females respectively). Although Australian guidelines present a singular recommendation for adults, only own-gender estimates were used as considerable gender differences persist in alcohol-related risk perceptions [28]. In analyses, we distinguished estimates of ≤ 2 standard drinks (i.e., below or consistent with the NHMRC long-term high-risk guidelines and therefore considered to not over-estimate alcohol-related risk) [27] from combined over-estimate/don’t know responses (i.e., over-estimated risk).

#### Harm minimisation strategies used when consuming alcohol

Frequency of use for four harm minimisation strategies when usually consuming alcohol were assessed (three existing items “Count the number of drinks you have”, “Deliberately alternate between alcoholic and non-alcoholic drinks”, “Limit the number of drinks you have in an evening due to driving” [18], in addition to original item, “Limit the number of drinks you have in an evening (e.g., to get up early for children, sport etc.)”). Responses were required on a 5-point scale, and collapsed to indicate strategies used at least sometimes (‘Always/Most of the time/Sometimes’ = “Yes” vs ‘Rarely/Never’ = “No”).

#### Reductions in alcohol consumption

Past-year reductions in alcohol consumption were reported in the survey using five dichotomous items (e.g., In the last 12 months have you… “Reduced the amount of alcohol you drink at any one time”), which represented reductions in both the volume and frequency of alcohol consumption, temporary and more permanent cessation, as well as substitution with lower-alcohol beverages. Participants were instructed to ‘tick all that apply’, with each item checked (as opposed to unchecked) in the survey form included as a positive response for analysis. Additional options ‘None of the above’ (selected by 40.0% of participants), ‘Don’t know/ unsure’ (4.0%), and ‘Prefer not to answer’ (0.2%) were provided; a response was required on at least one option of the measure.

#### Primary outcome: changing alcohol consumption behaviours because of energy-related concerns

Due to a dearth of existing research on changes in alcohol consumption because of energy-related concerns, two single-item outcome measures were developed to assess the usual frequency of this behaviour: When you have an alcoholic drink, how often do you… “Limit the number of drinks because you are concerned about the calories/kilojoules/effects on body weight”, and “Drink lower carb [carbohydrate] alcohol because you are concerned about the calories/kilojoules”. The latter item included reference to only energy content, as there is little evidence that links lower-carbohydrate (‘low-carb’) drink consumption with reduced body weight. Specific health claims such as weight loss cannot be made for alcoholic beverages under Australian law, except for nutrition content claims including on the basis of energy or carbohydrate [29]. As such, energy content is often emphasised in lower-carb alcohol promotions [30], and was assumed to be more salient to respondents than effects on body weight. Responses to the two items were required on a 5-point scale. They were strongly positively correlated (Pearson *r* = 0.68, *p* < 0.001*, n* = 801) and demonstrated good internal consistency (α = 0.81), so to distinguish participants who reported one or both of the behaviours at least sometimes (collapsed from ‘Always/Most of the time/Sometimes’ = “Yes”) from those who reported neither of the behaviours (both ‘Rarely/Never’ = “No”), a combined variable was used for analyses.

### Analyses

Frequency analyses explored sample characteristics. Harm minimisation strategies used when consuming alcohol, reductions in alcohol consumption, and motivations for reduction were compared by participant subgroups (those who had and had not changed alcohol consumption behaviours because of energy-related concerns) with Pearson’s chi-squared tests and Fisher’s Exact tests as appropriate. These analyses were stratified by gender, as gender differences are well-established in reducing (i.e., more frequently reported by men) and ceasing consumption behaviour (more frequently reported by women) [31].

To inform the predictor variables used in the modelling approach for each of the overall and gender-stratified samples, chi-squared tests were also used to assess the association between demographic and alcohol-related predictors, and the outcome measure of changing alcohol consumption behaviours because of energy-related concerns. Only significant predictors from univariate analyses in each of the respective samples were then entered simultaneously in multivariable binary logistic regression models (with those ‘Rarely/Never’ changing alcohol consumption behaviours because of energy-related concern as the reference category), which were used to determine the association between demographic and alcohol-related characteristics and the likelihood that participants changed alcohol consumption behaviour because of energy-related concerns. The multivariable model in the total sample included six demographic characteristics (gender, age group, remoteness, current employment status, household income, and parent status), in addition to risky alcohol consumption and frequency of alcohol consumption. The model for males included three demographic characteristics (age group, remoteness, current employment status) and frequency of alcohol consumption; these variables were also included for females, in addition to marital and parental statuses and risky alcohol consumption. All analyses were conducted in IBM SPSS Statistics Version 21 (IBM Corp, Armonk, NY, USA), and a two-sided Type I error rate of α = 0.05 was assumed for all significance tests.

### Missing data

Unless otherwise specified, ‘Don’t know’ or similar response options were not used for other measures. Listwise deletion was used where relevant; in addition to BMI data (described above), remoteness and socioeconomic status could not be derived from non-valid postcodes (*n* = 4, 0.5%). All other data were complete.

## Results

The sample (*n* = 801) comprised 50.2% females, with 46.6% aged 30–44 years and 41.1% aged 45–59 years (see Table 1 for further sample detail). Of participants with valid BMI data (*n* = 642), 43.6% were either underweight or in healthy weight range, and just over half (54.6%) considered themselves as either underweight or of acceptable weight. More than three-quarters (77.9%) of this sample of drinkers reported typically consuming alcohol at least weekly and 43.9% reported consumption above NHMRC long-term risk guidelines (average consumption of > 2 standard drinks/day). More than half (59.4%) either reported that they did not know or overestimated long-term low-risk levels of consumption (Table 1).

**Table 1:** Changing alcohol consumption behaviours because of energy-related concerns, and univariate associations with sample characteristics

**Changing alcohol consumption behaviours because of energy-related concerns**		Yes %	No %	
Total sample (*n* = 801)				
Changing alcohol consumption behaviours because of energy-related concerns^a^		*62.5*	*37.5*	
Limiting the number of drinks consumed, because of concern about the calories/kilojoules/effects on body weight		56.4	43.6	
Drinking lower carb alcohol, because of concern about the calories/kilojoules		46.4	53.6	
	Participants changing alcohol consumption behaviours because of energy-related concern			
**Demographic characteristics**	Total sample %	Yes %	No %	Difference between groups, *p*
Gender (*n* = 801)				**.001**
Female	50.2	68.2	31.8	
Male	49.8	56.9	43.1	
Age group (*n* = 801)				** < .001**
18–29	12.4	69.7	30.3	
30–44	46.6	69.2	30.8	
45–59	41.1	52.9	47.1	
Remoteness (*n* = 797)				** < .001**
Major cities	82.2	65.8	34.2	
Rural/remote	17.8	47.2	52.8	
Marital status (*n* = 801)				.053
Married/de facto	75.2	64.5	35.5	
Not married (separated/divorced/widowed/never married)	24.8	56.8	43.2	
Highest level of completed education (*n* = 801)				.237
Primary/high school	18.4	56.5	43.5	
Certificate/diploma	41.6	64.3	35.7	
Bachelor degree or higher	40.1	63.6	36.4	
Current employment status (*n* = 801)				** < .001**
Not in paid employment	22.6	50.3	49.7	
In paid employment	77.4	66.1	33.9	
Household income (*n* = 801)				**.022**
< $80,000	37.8	59.7	40.3	
$80,001–120,000	28.3	70.0	30.0	
> $120,000	33.8	59.4	40.6	
Area-level socioeconomic status (*n* = 797)				.738
Most disadvantaged	29.7	61.6	38.4	
Least disadvantaged	70.3	62.9	37.1	
Parent of children under 18 years (*n* = 801)				** < .001**
Yes	73.8	66.5	33.5	
No	26.2	51.4	48.6	
**Health-related characteristics**				
BMI (*n* = 642)				.260
Underweight/healthy weight	43.6	62.1	37.9	
Overweight	32.4	64.9	35.1	
Obese	24.0	56.5	43.5	
Perceived weight (*n* = 801)				.156
Underweight/acceptable weight	54.6	64.8	35.2	
Overweight or Don’t know/can’t say	45.4	59.9	40.1	
**Alcohol consumption characteristics**				
Average daily alcohol consumption (*n* = 801)				** < .001**
Above long-term risk guideline	43.9	69.6	30.4	
Within long-term risk guideline	56.1	57.0	43.0	
Frequency of alcohol consumption (*n* = 801)				** < .001**
Daily/weekly	77.9	66.0	34.0	
Monthly	22.1	50.3	49.7	
Estimate of safe alcohol consumption level (*n* = 801)				.168
Overestimate guideline/don’t know	59.4	64.5	35.5	
Consistent with/below guideline	40.6	59.7	40.3	

*Note*. ^a^ Yes on combined outcome = ‘Limiting the number of drinks consumed, because of concern about the calories/kilojoules/effects on body weight’ and/or ‘Drinking lower carb alcohol, because of concern about the calories/kilojoules’. Alcohol outcome variables were dichotomised to Yes (‘at least sometimes’; Always/Most of the time/Sometimes) vs No (Rarely/Never). Values are rounded to one decimal place and may not sum to 100%. Bold values indicate significance at *p*<.05

Table 1 also shows that 62.5% of the sample of drinkers reported changing their alcohol consumption behaviour ‘at least sometimes’ because of energy-related concerns. This included both those who reported limiting the number of alcoholic drinks consumed when drinking because of energy-related concerns (56.4%) and/or those who reported drinking lower-carb alcohol because of energy-related concerns (46.4%). Female respondents reported more often than men that they had changed alcohol consumption behaviour because of energy-related concerns overall (68.2% vs 56.9%; χ^2^(1) = 10.85, *p* = 0.001, *n* = 801) or had limited their number of drinks for this reason (61.9% vs 50.9%; χ^2^(1) = 9.97, *p* = 0.002, *n* = 801). However, drinking lower-carb alcohol for this reason did not differ by gender (49.3% of women vs 43.6% of men; χ^2^(1) = 2.57, *p* = 0.11, *n* = 801). (Individual item results by gender not shown in tables).

The proportion of participants reporting changing alcohol consumption because of energy-related concerns also differed by age group (Table 1), with younger participants reporting changing consumption more often (69.7% of 18–29 year olds and 69.2% of 30–44 year olds, compared to 52.9% of 45–59 year olds, *p* < 0.001). Those in major cities (65.8% vs 47.2%, *p* < 0.001), in paid employment (66.1% vs 50.3%, *p* < 0.001), with household income $80,001–120,000 (70.0% vs 59.4% and 59.7% for those above and below this amount respectively, *p* = 0.022), and with children under 18 years (66.5% vs 51.4%, *p* < 0.001) also reported changing consumption more often. Changing consumption was also reported more often by those consuming alcohol above the long-term risk guideline (69.6% vs 57.0% those drinking within guidelines, *p* < 0.001) and either daily or weekly (66.0% vs 50.3% of those consuming alcohol monthly, *p* < 0.001). The other characteristics were not significant univariate predictors of changing alcohol consumption in the full sample.

### Associations between harm minimisation strategies used, reductions in consumption and motivations for reduction because of energy concerns

As strategies to minimise harm, more than three-quarters reported counting the number of drinks consumed at least sometimes (75.2%), while 61.9% reported deliberately alternating alcohol with non-alcoholic drinks. Most also reported limiting their number of drinks at least sometimes either to drive (77.7%) or for reasons such as getting up early for sport or children (77.8%) when having an alcoholic drink in an evening. Just over half (55.8%) of the sample reported reducing alcohol consumption in at least one way during the past 12 months. The methods of reduction reported most frequently were reducing the amount of alcohol consumed in a single occasion (26.0%) and reducing the number of occasions at which alcohol was consumed (24.3%). Only 4.0% had stopped drinking completely (Table 2).

**Table 2:** Harm minimisation strategies, reduced alcohol consumption, and motivations, by changing consumption because of energy-related concerns

	Total sample %	Male participants changing alcohol consumption behaviours because of energy-related concerns			Female participants changing alcohol consumption behaviours because of energy-related concerns		
		Yes %	No %	Difference between groups, *p*	Yes %	No %	Difference between groups, *p*
**Harm minimisation strategies used when consuming alcohol**^**a**^	(*n* = 801)	(*n* = 227)	(*n* = 172)		(*n* = 274)	(*n* = 128)	
Limit the number of drinks in an evening for other reason (e.g., to get up early for children, sport etc.)	77.8	90.7	51.2	** < .001**	89.8	64.8	** < .001**
Limit the number of drinks in an evening due to driving	77.7	86.3	66.9	** < .001**	86.5	57.8	** < .001**
Count number of drinks consumed	75.2	87.2	56.4	** < .001**	84.7	58.6	** < .001**
Deliberately alternate between alcoholic and non-alcoholic drinks	61.9	76.7	31.4	** < .001**	78.8	40.6	** < .001**
**Reductions in alcohol consumption (past 12 months)**							
Any reduction item	55.8	67.8	34.3	** < .001**	67.9	37.5	** < .001**
Reduced amount per occasion	26.0	32.2	16.9	**.001**	29.9	18.8	**.018**
Reduced number of occasions	24.3	28.2	17.4	**.012**	29.6	15.6	**.003**
Stopped drinking for a period of time	16.9	16.3	11.0	.135	20.8	17.2	.395
Switched to low alcohol drinks	13.9	22.9	4.1	** < .001**	18.2	1.6	** < .001**^†^
Stopped drinking completely	4.0	6.2	0.6	**.003**^†^	4.0	4.7	.755

*Note*. ^a^Reported at least ‘sometimes’, when having an alcoholic drink. Bold values indicate significance at *p* < .05. ^†^Using Fisher’s Exact Test

Each harm-minimisation strategy was more often reported by male and female participants who had changed their alcohol intake due to energy-related concerns compared to those without such concerns (each *p* < 0.001; Table 2). As shown in Table 2, both males and females who reported they had changed their alcohol intake because of energy-related concerns (compared to those who had not) more often reduced the amount of alcohol consumed per occasion, reduced the number of occasions at which alcohol was consumed, and switched to low-alcohol drinks. They did not differ, however, in stopping drinking for only ‘a period of time’. Among males only, those who had changed their alcohol consumption behaviours because of energy-related concerns more often reported having stopped drinking completely (6.2% vs 0.6% of males who had not, *p* = 0.003). It was also more often that participants who had changed their alcohol consumption behaviours because of energy-related concerns (67.8% of males and 67.9% of females), compared to those who had not (34.3% and 37.5%, respectively), reported at least one of the items indicating reduced alcohol consumption (both *p* < 0.001).

### Factors associated with changing alcohol consumption because of energy concerns

As shown in Table 3, participants who were female (OR = 1.80, 95%CI = 1.30–2.49), aged 30–44 years (OR = 1.63, 95%CI = 1.16–2.28), resided in major cities (OR = 2.22, 95%CI = 1.50–3.29), reported household income of $80,001–120,000 (OR = 1.54, 95%CI = 1.03–2.28), typically drank alcohol above long-term risk guidelines (OR = 1.57, 95%CI = 1.13–2.20), and drank daily/weekly (OR = 1.59, 95%CI = 1.08–2.33) had increased odds of changing alcohol consumption behaviours because of energy-related concerns, while those who were not in current paid employment had reduced odds of doing so (OR = 0.54, 95%CI = 0.37–0.79).

**Table 3:** Demographic and alcohol consumption-related predictors of changing alcohol consumption behaviours because of energy-related concerns

Multivariate logistic regressions: Predictor variable	Total sample^*^ (*N* = 797)			Male participants^†^ (*n* = 397)			Female participants^‡^ (*n* = 400)	
	OR (95% CI)	*p*	OR (95% CI)		*p*	OR (95% CI)		*p*
**Gender** (R/C: male)								
Female	1.80 (1.30–2.49)	** < .001**	-			-		
**Age group **(R/C: 45–59)		**.010**			**.037**			.103
18–29	1.66 (0.99–2.79)	.055	1.86 (0.85–4.04)		.118	1.70 (0.83–3.51)		.149
30–44	1.63 (1.16–2.28)	**.005**	1.70 (1.09–2.64)		**.019**	1.68 (1.01–2.77)		**.044**
**Remoteness **(R/C: rural/remote*)*								
Major cities	2.22 (1.50–3.29)	** < .001**	1.76 (1.01–3.08)		**.046**	2.65 (1.53–4.58)		**.001**
**Marital status** (R/C: not married (separated/divorced/widowed/never married))	*NA*		*NA*					
Married/de facto						1.40 (0.83–2.38)		.211
**Current employment status** (R/C: in current paid employment)								
Not in paid employment	0.54 (0.37–0.79)	**.002**	0.47 (0.27–0.84)		**.011**	0.57 (0.35–0.94)		**.026**
**Household income **(R/C: > $120,000)		.104	*NA*			*NA*		
< $80,000	1.22 (0.83–1.79)	.303						
$80,001–120,000	1.54 (1.03–2.28)	**.033**						
**Parent status** (R/C: not a parent of children under 18 years)			*NA*					
Parent of children under 18 years	1.41 (0.99–2.02)	.056				2.39 (1.38–4.14)		**.002**
**Average alcohol consumption** (R/C: Not above long-term risk guideline)			*NA*					
Above long-term risk guideline	1.57 (1.13–2.20)	**.008**				2.68 (1.58–4.56)		** < .001**
**Frequency of alcohol consumption** (R/C: Monthly)								
Daily/weekly	1.59 (1.08–2.33)	**.018**	2.27 (1.31–3.94)		**.004**	1.17 (0.69–1.97)		.562

*Note. R*/C=Reference categories, where predictor included. OR=Odds ratio; CI=Confidence interval. *NA*=Predictor term not significant in univariate analyses, and not included in multivariable model. ^*^Model χ^2^ (10)=90.37, *p*<.001, *R*^2^ (Nagelkerke)=.15. ^†^Model χ^2^ (5)=33.51, p<.001, *R*^2^ (Nagelkerke)=.11. ^‡^Model χ^2^ (8)=55.96, p<.001, *R*^2^ (Nagelkerke)=.18. Bold values indicate significance at *p*<.05

Similarly, in the stratified models, males not in current paid employment had reduced odds of changing alcohol consumption behaviours because of energy-related concerns (OR = 0.47, 95%CI = 0.27–0.84), while those aged 30–44 years (OR = 1.70, 95%CI = 1.09–2.64), major city residents (OR = 1.76, 95%CI = 1.01–3.08), and those reporting daily/weekly alcohol consumption (OR = 2.27, 95%CI = 1.31–3.94) had increased odds of doing so. Female respondents not in current paid employment also had reduced odds of changing alcohol consumption behaviours because of energy-related concerns (OR = 0.57, 95%CI = 0.35–0.94), while odds were also higher for those aged 30–44 (OR = 1.68, 95%CI = 1.01–2.77), major city residents (OR = 2.65, 95%CI = 1.53–4.58), and those reporting average daily alcohol consumption above long-term risk guidelines (OR = 2.68, 95%CI = 1.58–4.56). Parent status (i.e., mothers of children under 18 years, OR = 2.39, 95%CI = 1.38–4.14) was also associated with changing alcohol consumption behaviours because of energy-related concerns in the multivariable model for females, while marital status and frequency of alcohol consumption were not (Table 3).

## Discussion

This study investigated changing alcohol consumption behaviours because of energy-related concerns, and co-occurring harm minimisation strategies and reductions in alcohol consumption, in a sample of 801 adult drinkers. A key finding was that a substantial proportion (62.5%) of respondents reported changing alcohol consumption due to concerns relating to the energy content of alcohol. Importantly, those who changed their alcohol consumption because of energy content concerns were also more likely to report having reduced their drinking in the past year. This association might imply that increased awareness of energy content may contribute to reduced alcohol consumption among adult drinkers, and that messaging to increase awareness of energy content is a potentially promising target for future population alcohol control interventions.

A key objective of the Australian National Alcohol Strategy 2019–2028 [32] is to improve understanding of the energy intake associated with alcohol consumption and potential weight gain. This has the potential to motivate behaviour change among drinkers. Health promotion efforts in this area need to be informed by evidence on the extent to which energy-related concern currently motivates alcohol consumption. The extent of self-reported engagement in changed drinking behaviour in the present sample indicates substantial consumer interest in modifying and reducing alcohol consumption for energy concerns. To date, limited information from Australian governments has highlighted the link between alcohol and increased energy consumption. The LiveLighter® public health education website, funded by the Western Australian Department of Health and delivered by Cancer Council WA, has developed information for one jurisdiction. There have been no nationally funded campaigns, and energy content listings on consumer alcohol products are not currently mandated [29]. However, markets around the world have seen rapid increase in the popularity of low-carb beers in particular [33], and the popularity of low-carb diets has likely also accelerated consumer uptake. Even low-carb but full-strength alcohol beverages may not be significantly lower in total energy than lower-alcohol beverages, given the greater energy density of pure alcohol compared to carbohydrate. Despite this, consumers may perceive low-carb drinks to be lower in alcohol [34]. Most low-carb beer drinkers believe that low-carb beer is healthier, and many (44%) believe it to be less fattening than light beer [35]. The alcohol industry may be capitalising on the low consumer awareness of energy content in alcohol, as well as these misconceptions about low-carb alcoholic beverages specifically, with heavy promotion of low-carb products, which are permitted under current Australian standards.

This study highlighted that certain subgroups are already more likely to be changing their alcohol consumption for energy-related concerns: women, city residents, those aged 30–44 years or with household income $80,001–120,000, and risky/more frequent drinkers had increased odds of changing consumption for energy-related concern; unemployed respondents had reduced odds of doing so. These sociodemographic subgroup results are comparable to findings overseas: for example, women and those with higher scores on an alcohol use disorder measure were more likely to report attempting to reduce consumption in England, including for weight loss [20]. There is currently an underutilised opportunity for health promotion communication and strategies including nutrition information labelling that may motivate more individuals in these subgroups to reduce their consumption, as few of these communication strategies currently exist. It is highly probable that motivated individuals are already more health conscious, as the association between changing alcohol consumption due to energy-related concerns and harm-minimisation strategies in the present study may suggest. This may reflect a desire to moderate alcohol consumption more generally, although more research (including qualitative exploration of these potential motivations) is required to confirm this. It is well-established that women are more likely to monitor energy intake, and engage in various harm minimisation strategies to reduce alcohol consumption [36]. Therefore, women may be more likely to change consumption with the aim of body weight maintenance or weight loss.

These results also indicate that changing consumption may be most prominent in those who are already drinking more frequently, and for the total and stratified female but not male samples, in those drinking at risky levels. We also found sociodemographic population segments that are less likely to be reducing consumption for energy-related reasons, including men, rural/remote residents, and older or unemployed people. Further research should assess whether population-wide or targeted messaging, highlighting the energy content in alcohol beverages, may motivate reduced, or lower-alcohol beverage, consumption in these groups. This is particularly important in Australia, where men and non-metropolitan residents are more likely to exceed alcohol guidelines than women and metropolitan residents, respectively [18].

Importantly, neither BMI nor perceived weight significantly predicted changing alcohol consumption in the full sample. While we acknowledge this is a complex area for intervention, people with higher BMI and/or higher perceived weight represent a key subgroup that could benefit from energy-related messaging because of the established links between alcohol consumption, higher BMI [7] and overweight and obesity [8]. Engaging this subgroup could potentially reduce healthcare system costs, morbidity, and mortality; further research to determine existing consumer awareness and motivations to change is a priority.

Future messaging could take several forms. Systems-based policy actions, such as nutritional/energy product (e.g., front-of-pack, menu) labelling have the long-term potential to reduce population alcohol consumption. Labelling interventions are frequently used to alter choice, and energy labelling specifically has been associated with decreased alcohol use [37]. Studies have found that increasing consumer understanding of food energy content through nutrition labelling reduces the amount of energy purchased and consumed [38]. Similarly, energy labelling on alcoholic products has been shown experimentally to reduce intended drinking [39], and data from food contexts are promising. However, the current evidence base on alcohol energy labels includes mainly studies of low methodological quality, with proxy outcomes other than of actual alcohol consumption, and a paucity of real-world (e.g., retail) settings [21].

Despite international public health calls [16, 40] and specific World Health Organization recommendations for widespread provision of nutrition information on packaged alcoholic products to guide informed consumer choice [41], energy labelling (i.e., the listing of calorie/kilojoule information) is not mandatory in many jurisdictions, including the United States, European Union, and Australia. Reports suggest international alcohol industry pressure has delayed more widespread uptake in international policy (e.g., the Codex Alimentarius) of labelling requirements supported by public health evidence [42]. Voluntary industry uptake of such labelling has been limited to date; 5.1% of alcoholic beverage containers showed nutritional data including energy information in a recent New Zealand audit [40], and earlier in England only 1.3% showed energy information [43]. Despite this, studies show strong public support for alcohol nutrition and energy labels, including among drinkers. Support for alcohol-related labelling and packaging policy (i.e., energy, ingredient, standard drink, health and pregnancy warnings) among Australian respondents in a recent cross-cultural study was higher than for price-based initiatives or advertising/sponsorship restrictions [44]. These responses may also have greater consumer acceptability than price-based interventions, despite the cost-effectiveness of price-based measures for alcohol control in general, and the projected effectiveness of increased alcohol costs for obesity prevention specifically [45]. Consumers’ right to health information on alcohol products [46] also reinforces the ethical importance of implementing energy labels. It has been suggested that this right should be prioritised, independent of information provision demonstrating effectiveness in changing behaviour [47, 48].

Recent international calls have been made for widespread alcohol energy labelling, including by key Australian health groups (e.g., Australian Chronic Disease Prevention Alliance, Cancer Council Australia, Foundation for Alcohol Research and Education), who advocate for alcohol labelling consistent with current food and non-alcoholic beverage requirements. The Australian National Preventive Health Strategy 2021–2030 specifies a policy achievement that consumer choice will be “guided by energy and ingredient labelling on all packaged alcoholic products” ([49] p. 54). Alcoholic products are within the scope of current Food Standards Australia New Zealand consultation to promote consumers’ informed healthy choice with improved labelling policy, recognising the limitations of current requirements. Similarly, a recent recommendation from the United Kingdom was that all alcohol product labels should include “ingredients and nutritional information such as calories” ([50] p. 46).

Such interventions combined can form powerful and persuasive messages. Media and education campaigns, highlighting the link between alcohol and weight gain, should also be considered. Alcohol advertising often uses health-related messaging and positions products including low-carb alcohol as a healthier choice [30]. To counter this, a focus on reducing total volume of alcohol consumption is required, which may involve lower-alcohol beverages (and therefore lower energy content) [33], if not the consumption of fewer beverages altogether. As similar volumes are consumed of lower-alcohol and standard alcoholic beverages, evidence shows that a switch to drinks with lower alcohol content typically represents a reduction in total alcohol consumption, rather than being compensated for with additional drinks [51].

### Limitations

Several limitations should be considered when interpreting these results. Firstly, this study used self-report measures, which may be subject to social desirability and other response biases, particularly for energy-related concern and alcohol use as potentially sensitive issues. Both alcohol consumption and energy intake are typically under-reported by survey respondents [52, 53], which may impact reliability. Further, prompted items were designed for the purposes of this exploratory survey, due to a dearth of existing tools on this outcome. Further research should investigate reliability and validity, and consider supplementing the energy-related concern items with more detailed measures of consumption and perceptions (e.g., food recall data with associated prompts, or dietary habit data). Respondents could change consumption due to energy-related concerns in ways not explicitly captured in these items. For example, respondents may also avoid drinking on particular days of the week or times of the year.

Further, height and/or weight data was not reported by a total of 19.1% participants, who were subsequently excluded from a univariate analysis using BMI. Whilst this level of missing data is comparable to other self-report surveys with large community samples of adults [54], we recognise that those not knowing or not willing to report this data may also systematically differ on health-related outcomes, and be an important subgroup for future focus.

Thirdly, recruitment was via non-probabilistic sampling, which limits population representativeness. A quota for parents with children under 18 years was also set for the broader study, which therefore over-sampled this group. Future studies could maximise representativeness with random sampling or demographic quotas consistent with national population characteristics.

## Conclusions

Overall, this study shows population segments motivated to reduce consumption due to the energy content in alcohol products. The market proliferation of lower-carb products may suggest that there is confusion about the energy content of these products and their role in reducing energy consumption, which requires further investigation. Given the association between alcohol consumption and weight gain, and that alcoholic beverages are the main contributor to the discretionary energy intake of Australian adults, policy responses such as nutritional labelling have potential to inform consumers. Further research should investigate the likely impact of messaging through educational campaigns as a harm prevention strategy for both alcohol use and obesity, and potential strategies to reduce alcohol consumption and disease burden among people with higher relative body weight.

## Data Availability

The dataset used and analysed during the current study is available from the corresponding author on reasonable request. The full text of study survey measures is available online via the Figshare repository, [https://doi.org/10.25451/flinders.19221279.v1].

## Abbreviations

BMI: Body mass index
CI: Confidence interval
NHMRC: National Health and Medical Research Council
